# Factors influencing women’s perceptions of choice and control during pregnancy and birth: a cross-sectional study

**DOI:** 10.1186/s12884-021-04106-8

**Published:** 2021-10-01

**Authors:** Patricia Leahy-Warren, Helen Mulcahy, Paul Corcoran, Róisín Bradley, Mary O’Connor, Rhona O’Connell

**Affiliations:** 1 School of Nursing and Midwifery, Brookfield Health Sciences Complex, College Road, Cork, T12 AK54 Ireland; 2 National Perinatal Epidemiology Centre, Cork University Maternity Hospital, Wilton, Cork, T12 DC4A Ireland

**Keywords:** Choice, Control, Maternity services, Midwifery, Childbirth, Models of maternity care

## Abstract

**Background:**

Women across the world value choice and control throughout their maternity care experiences. In response to this health policy and frameworks are adapting and developing. The concepts of choice and control are extrinsically complex and open to interpretation by healthcare professionals and service users, with the two not necessarily aligning. Depending on a number of factors, women’s experiences of choice and control within the same maternity care system may be very different. This study aimed to investigate the factors influencing women’s perceptions of choice and control during pregnancy and birth in Ireland.

**Methods:**

We conducted a cross-sectional study using an adapted version of the UK national maternity experience survey (National Perinatal Epidemiology Unit). During March – July 2017, a sample of 1277 women were recruited from the postnatal wards of three maternity units and a tertiary maternity hospital. Poisson regression was used to assess the association between twelve factors and a series of measures of the women’s perception of choice and control.

**Results:**

Most women reported not having choice in the model or location of their maternity care but most reported being involved enough in decision-making, especially during birth. Women who availed of private maternity care reported higher levels of choice and control than those who availed of public maternity care. This factor was the most influential factor on almost all choice and control measures.

**Conclusion:**

Most women experiencing maternity care in Ireland report not having choice in the model and location of care. These are core elements of the Irish maternity strategy and significant investment will be required if improved choice is to be provided. Availing of private maternity care has the strongest influence on a woman’s perceived choice and control but many women cannot afford this type of care, nor may they want this model of care.

**Supplementary Information:**

The online version contains supplementary material available at 10.1186/s12884-021-04106-8.

## Background

In many countries worldwide, maternity services are going through a period of evaluation and change. Health policy and frameworks are placing increasing emphasis on concepts such as choice and control. Whether this is a tokenistic attempt to present health services as person centred [1] or a genuine pursuit to implement a different model of care remains unclear. Nonetheless, it is evident that women across the world value care that incorporates these two concepts.

In Ireland, maternity care is provided under the Maternity and Infant Care Scheme, this free service consists of shared care between the woman’s GP and an obstetric led hospital service. Women with private health insurance may choose private obstetric care but will attend the public hospital for the birth. Most midwives are hospital based and work in antenatal, intranatal or postnatal areas [2, 3]. Apart from a few community midwifery services, postnatal community support is provided by public health nurses, who also provide a range of health-related services across the lifespan.

### Public maternity care in Ireland

In Ireland, recommendations from national reports and strategies indicate a need for a more biopsychosocial approach to maternity care to foster choice and control [2, 3] including that at least 20% of women, − have access to midwifery-led continuity of care, based on the Domino service model^1^ [3]. Recent health policies make a case for services to relocate to community settings, an approach which is thought to be more cost effective and lead to improvements in patient satisfaction through continuity of care(r) [4]. In recent years these recommendations have been actualised on a small scale in some hospitals; examples include the establishment of community based antenatal clinics and Domino schemes for some women living within allocated catchment areas [5]. Despite evidence supporting alternative models of maternity care, GP and obstetric led hospital care remains the predominant pathway of care available to childbearing women in Ireland [6, 7].

### Private maternity care in Ireland

Alongside the public system, private obstetric led care is popular amongst those who can afford the cost The level of this varies between hospitals but about a third of women access private care [8]. Women who avail of private services receive antenatal care from their chosen consultant obstetrician in a clinic style setting. During labour they are cared for in a public maternity hospital by a midwife employed by the public health service; the obstetrician is usually present for the birth. If complications arise care is managed by an obstetrician. Once discharged, women are visited by a public health nurse in the community and follow up postnatal appointment(s) are arranged with their chosen obstetrician. Despite women being significantly more likely to have an obstetric intervention irrespective of obstetric risk factors [8], private obstetric led care remains the only viable option for many women to ensure continuity of carer in Ireland.

### Documenting women’s perspectives of care

In 2017, a year after the publication of the National Maternity Strategy (NMS) [3] and a year before the Eighth amendment^2^ was repealed from the Irish Constitution, there was a sense that change was on the horizon. The need to greater understand women’s experiences of maternity care and to obtain baseline data for future comparison initiated a review of women’s experiences of maternity services in the South / South West of Ireland. As populations change and maternity services evolve, surveys have become increasingly popular tools for measuring women’s experiences of maternity care; informing health policy; and initiating improvements to the quality of care [9]. Experiences during the perinatal period have an impact on the future health of both parent and child, therefore making it a critical time for health providers to ensure service users have a positive experience [10]. This survey offers the first large scale insight into the experiences of women accessing maternity care in four Irish maternity units/hospital. This paper further examines the results of this survey, with the aim of revealing how women experience choice and control within Irish maternity services.

### The concept of choice

Choice involves the act of picking between two or more options based on relevant, balanced information, in the context of one’s own birth philosophy [11, 12]. Choice within maternity care is different from that of general healthcare and is often more scrutinised, as the decisions made will inevitably affect that of their fetus/unborn baby [13, 14]. Choice during the perinatal period is thought to be largely relational, meaning that the choices that are made by women occur in the context of their relationship with their baby and with their healthcare provider. The availability of informed choice does not rely independently on the availability of evidence-based information, but instead depends on an in depth discussion with a professional who they have had the opportunity to establish a relationship and build up a rapport [15].

The complexity involved in this decision-making process has meant that choice within maternity services is not clearly defined and is open to interpretation by service users and healthcare professionals; with the two not necessarily aligning. This can lead to women feeling pressured into the option deemed ‘least risky’ and ‘most sensible’ by their healthcare practitioner(s) [16] and can result in women feeling a lack of control and prevent actualisation of their choice [12].

### The concept of control

Like choice, control in maternity care is a concept open to interpretation without a conclusive definition [11]. Control in maternity services has been found to include decision making, access to information, personal security and physical functioning [17]. Feelings of control have many benefits, including increased childbirth satisfaction; positive childbirth experience; emotional wellbeing; and supporting the transition to motherhood [17]. Provision of information and involvement in decision making is dependent on a number of factors including: how embedded the procedure is in routine care; whether there is a policy for ensuring and obtaining documented consent and whether clinical policies or guidelines recommend its use [18]. Thus, procedures such as fetal monitoring, vaginal examination, blood tests and ultrasound scans are more likely to be adopted in a ‘uniform’ and ‘un-consulted’ decision making approach. This suggests that the more a procedure is considered routine, the less likely there is to be decision making processes fostering patient involvement [18].

Women from ethnic minorities experience an array of inequalities within Irish maternity care. These can include ineffective communication [19], inadequate 24-h access to properly trained interpreters [19, 20], and a lack of culturally sensitive care. Furthermore women from ethnic minorities are expected to conform to the existing system [21]. The Lancet Series on disrespectful and abusive maternity care recognises that these behaviours are often normalised in maternity services worldwide and can go unnoticed by providers, patients, or both. In order to achieve respectful maternity care, disrespectful and abusive care needs to be acknowledged as a symptom of a wider issue relating to power imbalances and inequities that exists within many maternity services [22]. To ensure quality in maternity care, the concepts of choice and control need to be protected and harboured within clinical environments.

### Problem statement

The concepts of control and choice are open to different interpretations. Depending on a multitude of factors, women’s experiences of choice and control within the same maternity care system may be very different. Race, age, socioeconomic factors, co-morbidities, and parity can all influence outcomes [23]. There is a paucity of research examining the factors that influence women’s perception of choice and control within Irish maternity services. Gaining a greater understanding of these factors is needed to facilitate a wider discussion about how maternity related health policy addresses issues relating equity of choice and control for women accessing maternity care in Ireland. This study aimed to investigate the factors that influence women’s perceptions of choice and control during pregnancy and birth in Ireland.

## Method

### Design

We conducted a cross-sectional study using an adapted version of the questionnaire from the UK national maternity experience survey [24]. This questionnaire was used as part of a larger study examining women’s experiences of maternity care in a region of Ireland [25, 26]. The survey was adapted to reflect the Irish maternity services at the time of data collection. Questions were rephrased to reflect Irish terminology in terms of, access to maternity care, anomaly scans and antenatal screening tests.

### Setting and participants

Participants were recruited from three maternity units and a tertiary level maternity hospital over a five-month period, March–July 2017. Recruitment was undertaken by research midwives who liaised with the midwifery manager of postnatal wards to identify women who met the inclusion criteria. All women on the postnatal wards who were 18 years or older and able to communicate in English were approached and given the opportunity to participate. This included women who had given birth to a live baby and those whose babies were admitted to the neonatal intensive care unit. Women were not approached if they or their baby were critically unwell or if they had experienced a pregnancy loss or stillbirth. The completed questionnaires were submitted prior to hospital discharge, the mean duration of postnatal stay for the women was 3.3 days. A total of 1774 women consented to participate and 1277 questionnaires were returned. Based on power analysis a sample size of 1091 was considered sufficient for the study but to minimise the bias associated with a convenience sample, the size of the sample was increased. Ethical approval for the study was obtained (ECM4(f)07/02/17) along with permission from the Directorate of the services.

### Data analysis

A data-coding framework was developed prior to data collection, and responses obtained from the questionnaire were entered into Excel and transferred to Stata version 15.1 for analysis. Double data entry was undertaken, and data were checked.

A range of twelve participant characteristics were identified as factors with potential to influence choice and control and of the total sample (*n* = 1277), 1176 women (92%) provided complete data on all twelve of these factors. To limit the effect of missing data on reported associations, data analysis was confined to these 1176 women. Univariable and multivariable Poisson regression analyses were used to investigate factors associated with the Control variables. As recommended by Zou (2004) [27], robust error variances were used. Factors with a *p*-value< 0.25 in the univariable analysis were eligible for inclusion in the multivariable analysis. For all factors investigated, the unadjusted and adjusted relative risks (RR) and 95% confidence intervals (95% CIs) are presented. Associations with a *p*-value < 0.05 in the univariable analysis were considered to be of interest and were reported. Considering there was a total of 12 measures of choice and control, independent associations in the multivariable analysis were reported as strongly associated with the outcome only if they had a *p*-value< 0.0042 (i.e. < 0.05/12).

## Results

### Characteristic of participants

Less than 40% of women who responded were primiparous, whilst 64% of women had at least one child previously. At least three quarters of women were over 30 years of age, had completed third-level education (82%), had a planned pregnancy (80%), were of Irish nationality (85%) and accessed public maternity care (76%) (Table 1). In terms of morbidities during pregnancy, 9% of women experienced high blood pressure; 7% of women experienced gestational diabetes; and 12% experienced emotional or mental health problems (Table 1). Finally, less than half of women had a vaginal delivery (47%), with others having an instrumental delivery (16%); a planned caesarean-section (24%); or an emergency caesarean-section (14%) (Table 1). The profile of the study participants with respect to age, type of maternity care and mode of delivery was similar to the population of women who gave birth in the four study hospitals during 2017 (Table 1).

**Table 1:** Participant characteristics with comparison to all maternities in 2017

	Participants *N* = 1176^a^ % (n)	All maternities^b^ *N* = 11,287% (n)
First baby		
No	63.6 (748)	
Yes	36.4 (428)	
Age group		
< 30 years	21.3 (250)	26.5 (2988)
30–34 years	35.0 (412)	36.3 (4096)
35–39 years	34.9 (411)	30.7 (3469)
40+ years	8.8 (103)	6.5 (734)
Third level education		
No	17.8 (209)	
Yes	82.2 (967)	
Planned on becoming pregnancy with this baby		
No	19.8 (233)	
Yes	80.2 (943)	
Type of maternity care		
Public	76.1 (895)	82.2 (9280)
Private	23.9 (281)	17.8 (2007)
Nationality		
Non-Irish	14.8 (174)	
Irish	85.2 (1002)	
Experienced high blood pressure during pregnancy		
No	91.1 (1071)	
Yes	8.9 (105)	
Experienced gestational diabetes during pregnancy		
No	92.6 (1089)	
Yes	7.4 (87)	
Experienced any emotional or mental health problems during pregnancy		
No	87.7 (1031)	
Yes	12.3 (145)	
Mode of delivery	*N* = 1086	
Vaginal delivery	47.1 (512)	51.5 (5818)
Instrumental vaginal delivery	15.6 (169)	16.8 (1894)
Planned Caesarean section	23.6 (256)	18.7 (2109)
Emergency Caesarean-section	13.7 (149)	13.0 (1466)

^a^unless otherwise stated; ^b^all women who gave birth in the four study hospitals in 2017

### Choice and control

Results for choice and control are presented under the following headings: Women’s Experiences of Choice; Women’s Experiences of Control; Women’s Experiences of Involvement in Decision Making. Table 2 provides a summary of these results.

**Table 2:** Women’s reported perceptions of choice and control during antenatal care and childbirth

	Yes % (n)
1. Was offered choice of practitioner carrying out antenatal check-ups (*n* = 1078)	22.1% (238)
2. Was offered choice of where antenatal check-ups would take place (*n* = 1075)	31.6% (340)
3. Had choice of midwifery-led care (*n* = 805)	33.2% (267)
4. Had choice of Domino Scheme (*n* = 804)	14.6% (118)
5. ‘Always’ had time to discuss pregnancy during antenatal check-ups (*n* = 1145)	79.0% (905)
6. Felt you had a choice of having scans (*n* = 1116)	67.7% (756)
7. ‘Always’ involved enough in decisions about your antenatal care (*n* = 1162)	64.4% (748)
8. Was offered a choice of birth location (*n* = 1117)	15.6 (175)
9. Felt in control ‘almost always’ during childbirth (*n* = 1063)	29.6% (315)
10. Received pain relief at the time you wanted (*n* = 660)	81.8% (540)
11. ‘Always’ was involved enough in decisions about your care during labour and birth (*n* = 1111)	77.0% (856)
12. Felt under pressure from healthcare professional on decisions taken (*n* = 1076)	14.7% (158)

#### Women’s experiences of choice

One third of women (33.2%) had a choice of midwifery-led care and 14.6% reported access to the Domino service, which provides continuity of care, and only 2.3% reported being offered a choice of giving birth at home (Supplementary File 1 Table A). In terms of being offered Midwifery Led Care (Domino service), univariable analysis found that women with third level education and women with public maternity care were more likely to be offered this model of care (Supplementary File 1 Table C). Third level education (*p* = 0.004) and type of maternity care (*p* < 0.001) remained strongly associated in the multivariable analysis. In terms of choice of the Domino scheme, being a first-time mother was strongly associated with being offered this as an option (p < 0.001) (Supplementary File 1 Table D).

According to univariable analysis, older women (*p* = 0.018), women with private care (*p* = 0.011) and women who experienced gestational diabetes (0.019) were less likely to be offered choice about where their check-ups would take place but these variables were not strongly associated factors following multivariable analysis (Supplementary File 1 Table B).

Based on univariable analysis, women with third level education (*p* = 0.005), private care (*p* < 0.001), planned pregnancies (*p* = 0.008) and older women (*p* = 0.002) were more likely to be offered choices of practitioner carrying out check-ups (Table 3). Type of maternity care (*p* < 0.001) was the only variable to be strongly associated following multivariable analysis, with those who had private care being more likely to be offered the choice of practitioner (Table 3).

**Table 3:**
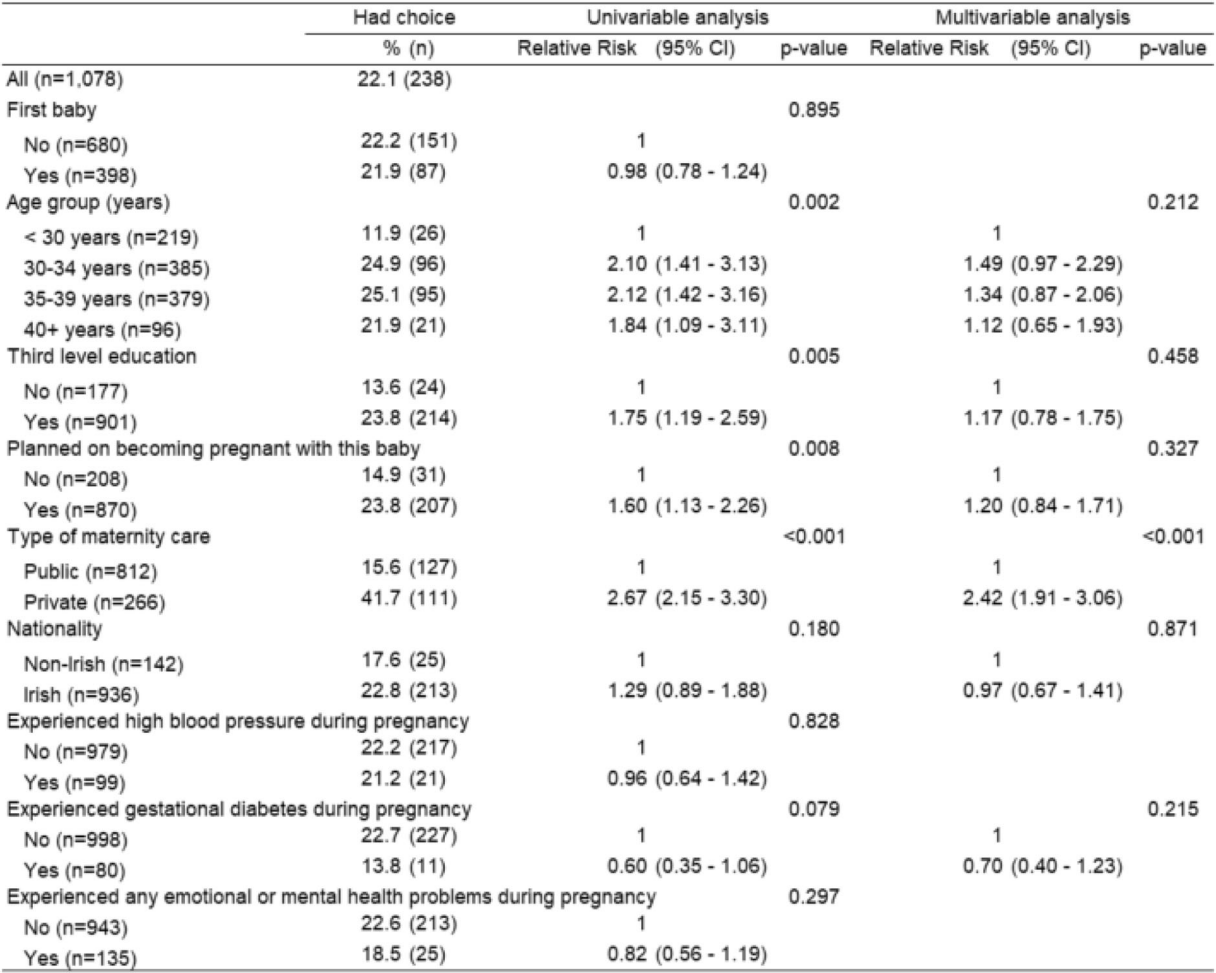
Women’s choice of practitioner carrying out check-ups and factors influencing their perception of this choice

Univariable analysis found that women who planned on becoming pregnant (*p* = 0.034), had private care (p < 0.001), identified as Irish (*p* = 0.021) and older women (*p* = 0.004) were more likely to have reported that they had choice about having antenatal scan(s) (Table 4). Following multivariable analysis, type of maternity care was the only strongly associated factor, meaning women with private care were more likely to answer that they had a choice of having scan(s).

**Table 4:**
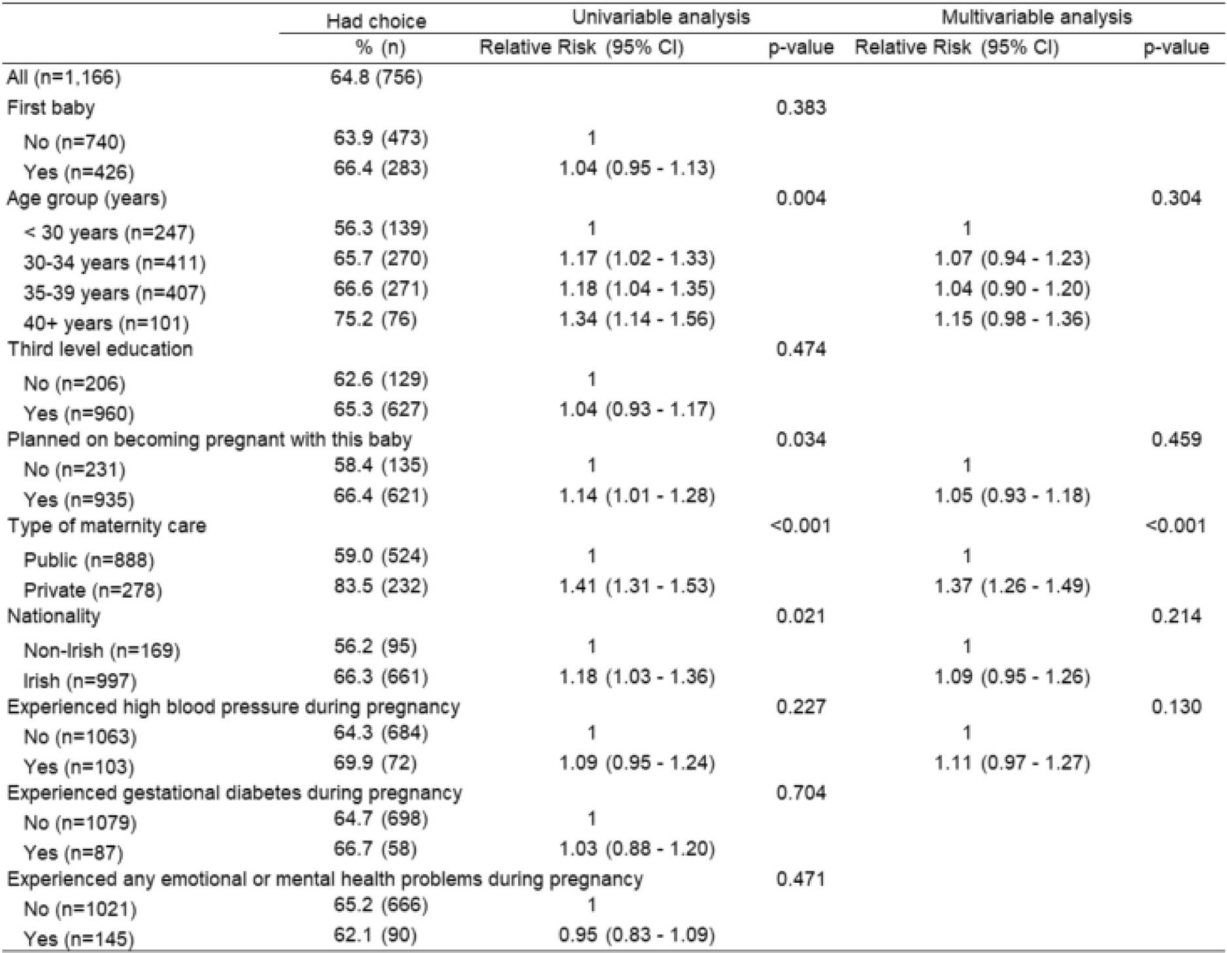
Women’s choice of scan(s) and factors influencing their perception of this choice

#### Women’s experiences of control

During antenatal check-ups, women were more likely to report always having enough time to ask questions or discuss their pregnancy if they were aged < 30 years (*p* = 0.009), had planned on becoming pregnant (*p* = 0.017), were receiving private care (*p* < 0.001) and did not experience emotional or mental health problem during pregnancy (*p* = 0.021) (Supplementary File 1 Table E). Following multivariable analysis, type of maternity care was the only variable to be strongly associated (*p* < 0.001), meaning women who received private care were more likely to answer, ‘yes always’ (Supplementary File 1 Table E).

Univariable analysis found that women who did not have third level education (*p* = 0.006) and women who identified as non-Irish (p < 0.001) were more likely to report being almost always in control during childbirth but only the latter was strongly associated based on the multivariable analysis(Table 5).

**Table 5:**
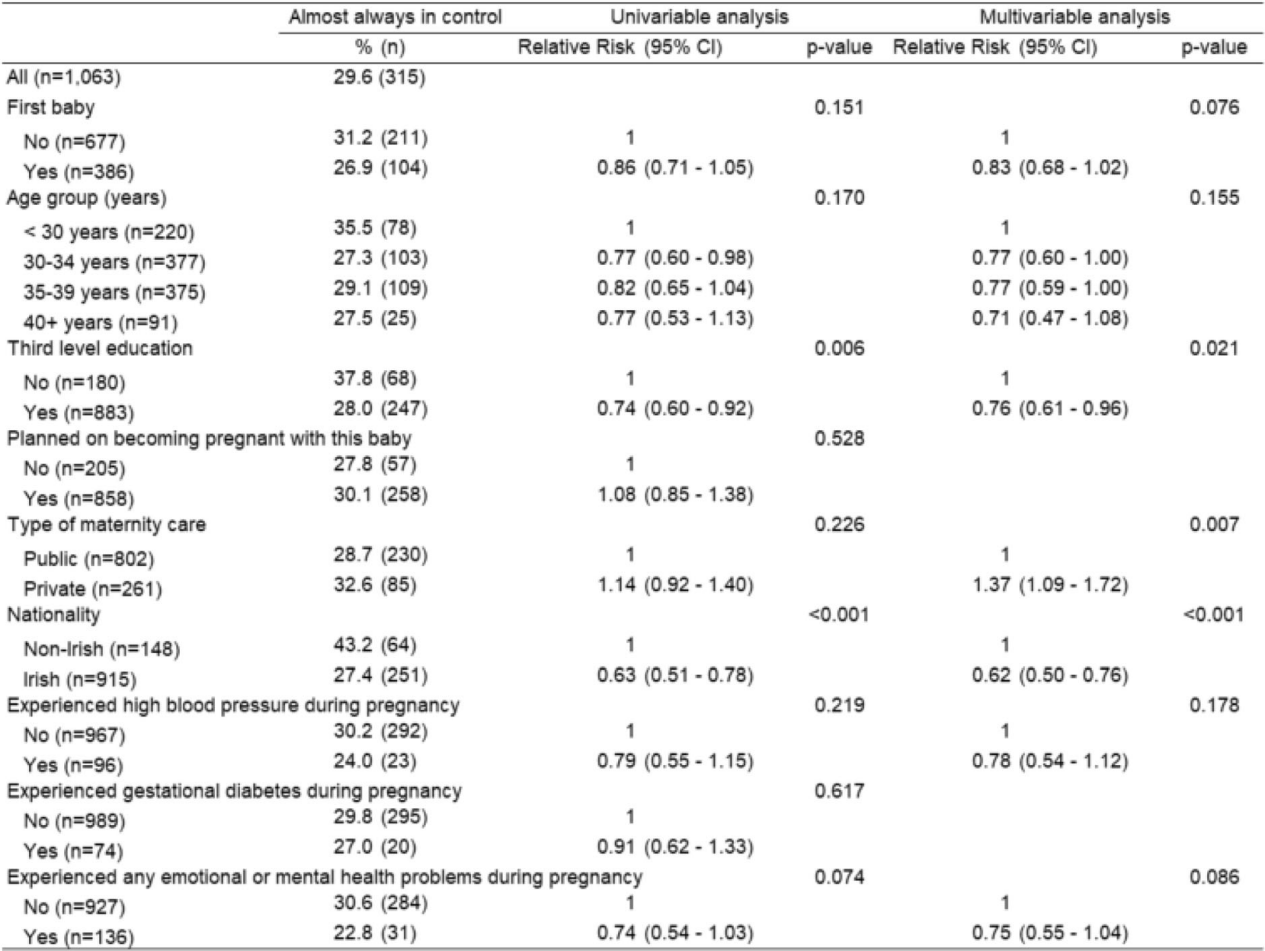
Women’s perception of control during childbirth and factors influencing this perception

None of the variables were associated with women’s perception of receiving pain relief at the ‘time wanted’ (Supplementary file Table H), suggesting that despite different characteristics, women had similar experiences of receiving pain relief at the ‘time wanted’.

#### Women’s experiences of involvement in decision making

Decision making is a fundamental component of feeling in control and for most women the involvement in decision making has a positive impact on their childbirth experience [6]. To further explore control this study examined women’s experiences of involvement in decision making. Univariable analysis found that women who were aged < 30 years (*p* = 0.002); had a planned pregnancy (*p* = 0.022); with private maternity care; were Irish; or did not experience any emotional or mental health problems during pregnancy were more likely to report feeling involved in decisions about their care during the antenatal period (Supplementary File 1 Table F). Multivariable analysis found that type of maternity care (*p* < 0.001) and experiencing any emotional or mental health problems during pregnancy (*p* = 0.003) remained strongly associated. Women with private care and women who did not experience any emotional or mental health problems during pregnancy were more likely to always feel involved in decisions about their antenatal care (Supplementary File Table F).

With regards to decision-making during childbirth, univariable analysis found that women with private care were more likely to report feeling involved enough (*p* = 0.003) but this was not strongly associated based on the multivariable analysis (Supplementary File 1 Table G).

Finally, women were asked if they felt under pressure from healthcare professionals (HCP) during decision making. Those who had an unplanned pregnancy (*p* = 0.011); public care (*p* = 0.004); high blood pressure during pregnancy (*p* < 0.001); experienced any emotional or mental health problems during pregnancy (*p* = 0.009); or who were non-Irish (p < 0.001) were more likely to have felt pressure from HCP during decision making (Table 6). Multivariable analysis found that nationality (p < 0.001) was the only variable to remain strongly associated, with non-Irish women were more likely to have felt pressure from HCP on decisions taken (Table 6).

**Table 6:**
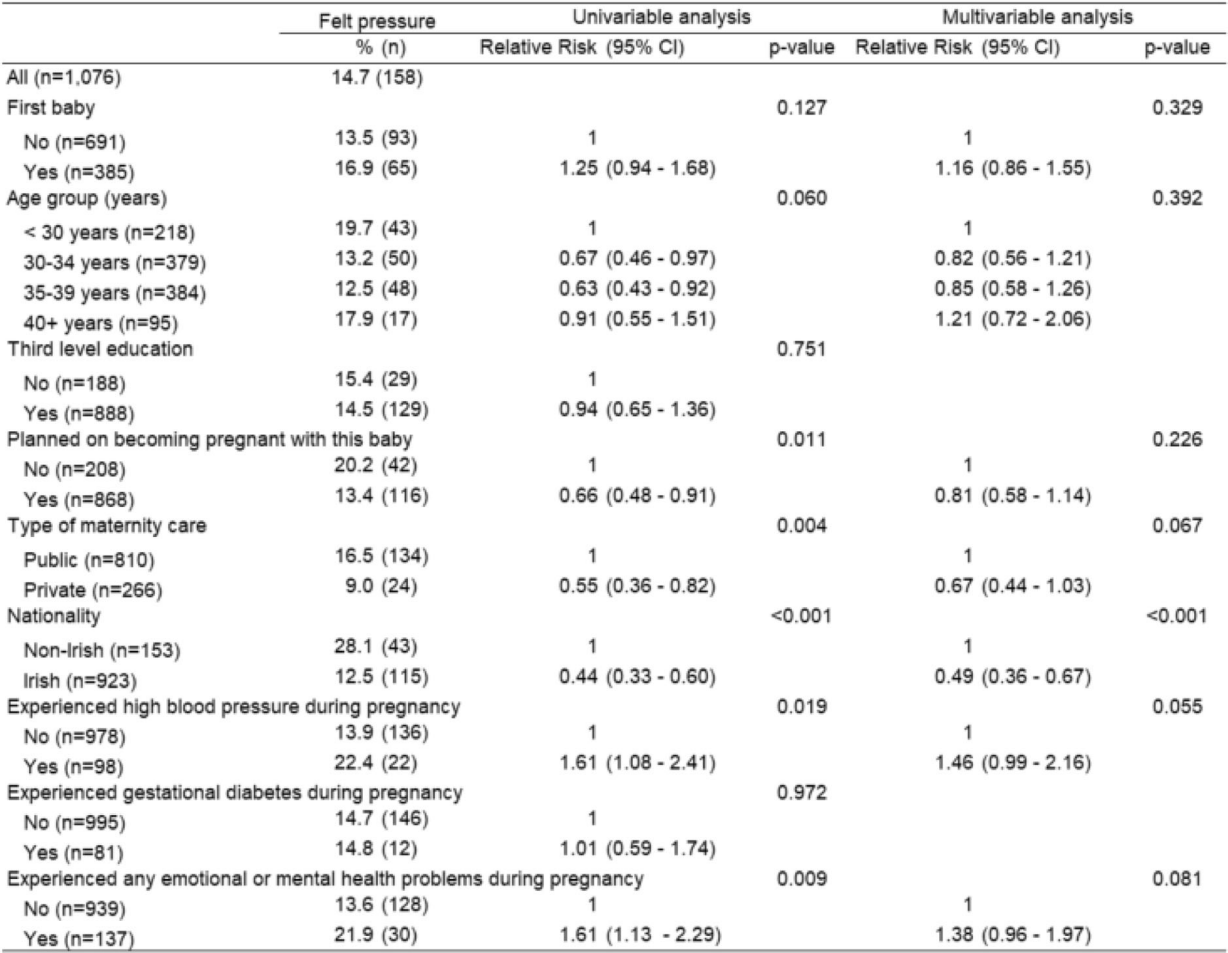
Women’s perception of feeling pressure from a healthcare professional during decision making and factors influencing this perception

## Discussion

### Demographics

Although a high number of the study participants had completed third level education (82.2%), their profile with respect to age and type of maternity care was similar to the population of women who gave birth in the four study hospitals during the study period.

### Mode of birth

The mode of birth is associated with different outcomes at 3 months postpartum. Women who had forceps assisted birth or unplanned caesarean section reporting the poorest health and wellbeing, whilst women’s physical and emotional health was least affected for those who had an unassisted vaginal birth or planned caesarean section [28]. The mode of birth was similar to the population of women who gave birth in the four study hospitals during the study period. The rates of caesarean sections (planned and emergency) were 37%, this is considerably higher than 10%, the amount deemed by the World Health Organisation (WHO) as necessary to reduce maternal and neonatal mortality. According to the WHO, caesarean section rates that increase above 10% are not associated with reductions in maternal or neonatal mortality rates, and could instead cause unnecessary, significant and permanent complications [29]. A recent Irish study, found that women in private care were more likely to give birth by caesarean section [6].

### Women’s experiences of choice and control

The only area whereby women were found to have equal access to choice was in relation to pain relief. This result is unsurprising as the vast majority of women gave birth in hospital, with access to pharmacological pain relief methods and the medical professionals needed to administer such methods (e.g. on-call anaesthetists for the administration of regional analgesia).

Women who reported unplanned pregnancy were more likely to experience a lack of choice in relation to choice of scans, practitioner, and access to the Domino scheme. They also perceived a lack of control in antenatal check-ups; being less likely to be involved in decision making and more likely to have felt pressure from a Health Care Worker (HCW). In Ireland, a report found that 16% of all pregnancies experienced by women were described as a ‘crisis pregnancy’ [30] and a 2015 study found that younger women, married women and women with lower educational achievement were at an increased risk of crisis pregnancy [31]. Although not all unplanned pregnancies could be considered a ‘crisis pregnancy’, research suggests that unplanned pregnancy is associated with an increased risk of postnatal depression at 9 months postpartum, particularly amongst women who felt unhappy or ambivalent towards their pregnancy [32].

The age of women was found to be another influential factor, with older women being more likely to have choice in relation to scans and antenatal check-up location. Whilst younger women were more likely to have reported feeling in control during antenatal check-ups and involved in decisions about their care. In Ireland, women who are older, are frequently considered high risk, which may account for their increased access to medical support (e.g. scans). This is an important factor to note as the average age of births to mothers in Ireland is currently 32.2 years, with 6.4% of mothers 40 years or over, these figures have increased steadily in the past two decades [33].

Women with third level education were more likely to be offered choice of practitioner and more likely to be offered choice of MLC. However, they were likely to feel less ‘in control’ in comparison to women who did not have third level education. These results are in contrast with similar studies internationally which found that with decreasing socio-economic position (including lower level of education and younger age) women were generally more likely to report not being treated respectfully or spoken to in a way that they could understand [34]. A hypothesis for these results may be that women who had a higher level of education were more likely to engage with educational material outside of Irish hospital sources, thus making them more aware of the options available within maternity services worldwide (e.g. the option to have a waterbirth in UK or Australia). Further research is needed to understand the experiences of women from different socioeconomic groups from an Irish perspective.

Non-Irish women were more likely to report being in control during childbirth ‘almost always’. The number of non-Irish women who took part in this study was 10% lower than the national figure. This combined with the limited research available nationally, examining how non-Irish women experience maternity care, means it is difficult to draw a conclusion at this time. Secondary analysis of a service user experience survey conducted in the UK, found that women from ethnic minority groups were less likely to feel spoken to so that they could understand, be treated with kindness, be sufficiently involved in decisions about their care and have confidence and trust in the staff [35]. As the number of non-Irish women accessing Irish maternity services increases, further research is needed to understand their experiences and guide maternity services provision, health policy and professional education to address migrant women’s individualised needs [36].

Women with pregnancy related complications (e.g. gestational diabetes or/and raised blood pressure) were less likely to experience choice and control. Women with gestational diabetes and women with raised blood pressure both reported feeling under pressure by healthcare professionals during decision making. Furthermore, women with gestational diabetes were less likely to be offered choice of where their antenatal checks ups would take place, whilst women with a raised blood pressure were less likely to have a choice to avail of Domino scheme. These results align with the Irish context where women who are categorised as high risk have limited choices regarding the type of maternity care they receive e.g. women must be deemed ‘low risk’ to access the Domino scheme [3]. Although there are studies looking at pregnant women’s experience of gestational diabetes and raised blood pressure, they do not often examine factors such as choice and control, as it is widely accepted in Ireland that choices within the medical model of care become more limited when complications arise.

Women who experienced emotional or mental health problems during pregnancy did not report a lack of choice in maternity care. However, they were found to perceive a lack of control in relation to the decision making during antenatal care and reported more pressure from HCW. These results are of importance, as it has been reported that women who experience birth trauma perceive lack of control and being subjected to authoritarian decision making [37]. One study found that despite women with mental health difficulties receiving substantially more care, they were significantly more worried about labour and had lower satisfaction with their birth experience [38]. The launch of the ‘Model for Perinatal Mental Health Services in Ireland’ [39], underpinned by a multidisciplinary team approach, is designed to offer individualised support to women experiencing mild to severe mental difficulties. It is envisaged that their needs will be responded to sensitively within maternity services [39]. Future research is required to determine whether such changes to maternity care provision are effective at alleviating the inequalities women experiencing mental health difficulties encounter during the perinatal period.

Type of maternity care was found to impact nine of the ten examined areas of choice and control. Women who had private care were more likely to have experienced choice and control throughout their maternity care, for example they were more likely to have choice of scans, more likely to felt involved in decision making, and more likely to have felt in control. It is assumed that this cohort of women had already chosen to have obstetric led care privately, therefore it was expected that they would report having less choice of midwifery led care and access to the Domino scheme as this is only offered in the public maternity care system. In contrast women who received public care were more likely to have felt under pressure from their healthcare provider when decision making. As women who access private care in Ireland are mainly cared for during birth and the postnatal period by midwives or public health nurses employed under the public health system within a public hospital, it would be reasonable to assume that the differences between their care is minimal. However, these results demonstrate the inequality that exists between women who access private and public maternity care in Ireland.

Finally, this study findings demonstrates the lack of birth place choices women have in the South/South West of Ireland, with over three quarters of women not being offered any birth place choices; 8.7% of those reporting that they had no birth place choices due to medical reasons and 10.8% of those stating they ‘did not know’ if they had been offered birth place choices. One of the maternity units in this study provides a home birth service within a specific catchment area, whilst in all other areas the homebirth service is predominantly provided by self-employed community midwives (SECM) on behalf of the Health Service Executive (HSE). Although nationally the number of home births is low at 0.3%, 58.8% of all homebirths nationally occur in the region where this study took place [40]. Despite this only 2.3% of women in the study reported being offered a choice of giving birth at home. The eligibility and suitability of homebirth for women is assessed under the HSE Policy [41]. Women who wish to give birth at home must meet certain criteria and be assessed by a consultant obstetrician in order to avail of the homebirth service. Studies have found that the interpretation of risk and the decision as to where to give birth is deeply subjective, with high risk women who wish to give birth at home perceiving risk differently to those who wish to give birth in hospital [42]. At the time of writing this paper, despite this being a recommendation of the 2016 National Maternity Strategy [3], there is no ‘alongside’ or ‘standalone’ birth centre in the South/South West of Ireland. It is clear that the vast majority of women in this study were not consulted about their birthplace choices and an implicit expectation existed for them to give birth in hospital.

### Limitations

This paper reports results from a larger study which did have some limitations, as with other surveys there was more responses from women of certain demographic groups. Women who responded were more likely to be older, living with their husband/partner and had completed third level education. During the five-month recruitment period, the number of eligible women approached was not recorded so a response rate could not be calculated. Use of a convenience sample is acknowledged as a limitation and future research on a national level using randomisation sampling is imperative to identify national needs and the provision of an evidence-based service delivery response. Although the sample represents women in the study region, the results cannot be generalised beyond this setting.

Finally, satisfaction surveys have been found to have important limitations which need to be considered when examining the results of this paper. The often-cited paper by van Teijlingen et al. (2003) [43] highlights that although satisfaction surveys often find that women are overwhelming satisfied with their maternity care, it is important to remember that marginal groups are rarely well represented in these surveys and service users tend to value the status quo over innovations of which they have no experience.

## Conclusion

From this study it is evident that women face unequal access to choice and control in Irish maternity services. Women have limited options in terms of birthplace choices with an implicit expectation on most of them to give birth in a hospital setting.

Women’s perceptions of choice and control is particularly impacted by the type of care they access; with women who access private care being more likely to experience choice and control in comparison to those who access public care. However, many women cannot afford this type of care or may prefer an alternative model of care, such as midwifery led care.

In addition to this woman who experience an unplanned pregnancy; emotional or mental health difficulty during pregnancy; morbidities during pregnancy and are non-Irish are more likely to have poorer experiences of choice and control. To conclude women’s perceptions vary depending on a number of variables, demonstrating that women do not have equitable access to choice and control in Irish maternity services.

## Supplementary Information

**Additional file 1.** Table A Women’s birth-place choices. Table B Women’s choice of where check-ups would take place and factors influencing their perception of this choice. Table C Women’s choices of midwifery-led care and factors influencing their perception of this choice. Table D Women’s choice of the DOMINO Scheme and factors influencing their perceptions of this choice. Table E Women’s perception of having enough time to ask questions or discuss pregnancy during antenatal check-ups and factors influencing this perception. Table F Women’s perception of being involved in decisions about their care during antenatal check-ups and factors influencing this perception. Table G Women’s perception of being involved in decisions about their care during labour and birth and factors influencing this perception. Table H Women’s perceptions of receiving pain relief at the time they wanted and factors influencing this pereception.

## Data Availability

Public access was not part of the ethical approval for this research study. If someone wants to request the data from this study, please contact the first author (Patricia Leahy-Warren) at patricia.leahy@ucc.ie

## Abbreviations

CSO: Central Statistics Office
D&A: Disrespectful and Abusive Treatment
DOH: Department of Health
DOMINO: Domiciliary In and Out
HCP: Healthcare Professional
HIQA: Health Information and Quality Authority
HPO: Healthcare Pricing Office
HSE: Health Service Executive
MBRRACE: Mothers and Babies: Reducing Risk through Audits and Confidential Enquiries
MLC: Midwifery-Led Care
NPEU: National Perinatal Epidemiology Unit
NMS: National Maternity Strategy
RR: Relative Risk
SECM: Self-Employed Community Midwives
SSWHG: South/South West Hospital Group
VIF: Variance Inflation Factor
WHO: World Health Organisation

## Footnotes

1 A ‘Domino’ scheme is available in some hospitals for women with low risk pregnancies within a defined geographical area (close to the maternity hospital). The scheme provides continuity of care by a team of midwives during pregnancy, labour and birth. Women may avail of early postnatal discharge with follow up community care by the midwifery team.

2 Eighth Amendment: A constitutional amendment which equated the life of the woman to the life of the fetus. This amendment prevented women from making many decisions during pregnancy and birth e.g. termination of pregnancy; birthplace choices; access to/refusal of medical treatment.
