# Supplementary material for: Factors influencing women’s perceptions of choice and control during pregnancy and birth: a cross-sectional study
Source: BMC Pregnancy Childbirth. 2021 Oct 1;21:667. doi: 10.1186/s12884-021-04106-8 (PMC8487111; doi:10.1186/s12884-021-04106-8)
Supplement: Supplementary file 1 — Additional file 1. Table A Women’s birth-place choices. Table B Women’s choice of where check-ups would take place and factors influencing their perception of this choice. Table C Women’s choices of midwifery-led care and factors influencing their perception of this choice. Table D Women’s choice of the DOMINO Scheme and factors influencing their perceptions of this choice. Table E Women’s perception of having enough time to ask questions or discuss pregnancy during antenatal check-ups and factors influencing this perception. Table F Women’s perception of being involved in decisions about their care during antenatal check-ups and factors influencing this perception. Table G Women’s perception of being involved in decisions about their care during labour and birth and factors influencing this perception. Table H Women’s perceptions of receiving pain relief at the time they wanted and factors influencing this pereception. [file 12884_2021_4106_MOESM1_ESM.docx]

**Supplementary File 1**

Table A Women’s birth-place choices

Table B Women’s choice of where check-ups would take place and factors influencing their perception of this choice

Table C Women’s choices of midwifery-led care and factors influencing their perception of this choice

Table D Women’s choice of the DOMINO Scheme and factors influencing their perceptions of this choice

Table E Women’s perception of having enough time to ask questions or discuss pregnancy during antenatal check-ups and factors influencing this perception

Table F Women’s perception of being involved in decisions about their care during antenatal check-ups and factors influencing this perception

Table G Women’s perception of being involved in decisions about their care during labour and birth and factors influencing this perception

Table H Women’s perceptions of receiving pain relief at the time they wanted and factors influencing this pereception
